# Combined Toxic Effects of BPA and Its Two Analogues BPAP and BPC in a 3D HepG2 Cell Model

**DOI:** 10.3390/molecules28073085

**Published:** 2023-03-30

**Authors:** Martina Štampar, Tim Ravnjak, Ana-Marija Domijan, Bojana Žegura

**Affiliations:** 1 Department of Genetic Toxicology and Cancer Biology, National Institute of Biology, 1000 Ljubljana, Slovenia; 2 Biotechnical Faculty, University of Ljubljana, 1000 Ljubljana, Slovenia; 3 Department of Pharmaceutical Botany, Faculty of Pharmacy and Biochemistry, University of Zagreb, 10000 Zagreb, Croatia

**Keywords:** BP analogues, hepatic in vitro 3D cell model, combined exposure, viability, oxidative stress

## Abstract

Bisphenol A (BPA) is one of the most commonly used substances in the manufacture of various everyday products. Growing concerns about its hazardous properties, including endocrine disruption and genotoxicity, have led to its gradual replacement by presumably safer analogues in manufacturing plastics. The widespread use of BPA and, more recently, its analogues has increased their residues in the environment. However, our knowledge of their toxicological profiles is limited and their combined effects are unknown. In the present study, we investigated the toxic effects caused by single bisphenols and by the combined exposure of BPA and its two analogues, BPAP and BPC, after short (24-h) and prolonged (96-h) exposure in HepG2 spheroids. The results showed that BPA did not reduce cell viability in HepG2 spheroids after 24-h exposure. In contrast, BPAP and BPC affected cell viability in HepG2 spheroids. Both binary mixtures (BPA/BPAP and BPA/BPC) decreased cell viability in a dose-dependent manner, but the significant difference was only observed for the combination of BPA/BPC (both at 40 µM). After 96-h exposure, none of the BPs studied affected cell viability in HepG2 spheroids. Only the combination of BPA/BPAP decreased cell viability in a dose-dependent manner that was significant for the combination of 4 µM BPA and 4 µM BPAP. None of the BPs and their binary mixtures studied affected the surface area and growth of spheroids as measured by planimetry. In addition, all BPs and their binary mixtures studied triggered oxidative stress, as measured by the production of reactive oxygen species and malondialdehyde, at both exposure times. Overall, the results suggest that it is important to study the effects of BPs as single compounds. It is even more important to study the effects of combined exposures, as the combined effects may differ from those induced by single compounds.

## 1. Introduction

Bisphenols are a group of synthetic chemicals used to produce polycarbonate plastics, epoxy resins, and thermal paper [1,2,3]. Among them, bisphenol A [4,4′-(propane-2,2-diyl)diphenol) (BPA)] is still the most frequently used to manufacture a range of everyday household items, such as baby bottles, food containers, paper products, and kitchen utensils [4,5,6]. BPA is an additive and can leach under various conditions from products such as toys and, more importantly, from bottles and containers into water and food. The general population is exposed to BPA daily through ingestion, inhalation, or dermal contact. It is, therefore, ubiquitous in the environment [7,8,9,10,11]. While many synthetic substances, including BPA, were initially considered harmless, scientists have discovered their adverse effects on human health and the environment over time. Reports from the late 1990s link BPA to various adverse effects, particularly disruption of the human endocrine system [12,13,14,15]. In addition, BPA has been shown to have genotoxic properties [16,17,18,19,20], can affect the reproductive, cardiovascular, immunological and respiratory systems, can induce tumours [21,22,23,24,25] and can impair cognitive and behavioural development [26]. Due to safety concerns, the European Union (EU) has restricted the use of BPA to protect human health and the environment [27]. The U.S. Food and Drug Administration (FDA) banned the use of BPA in baby cups, bottles, and infant formula packaging in 2012 and 2013, respectively, marking the beginning of regulatory action against the chemical [28]. In 2017, the European Chemical Agency (ECHA) added BPA to the Candidate List of Substances of Very High Concern [29] and the European Food Safety Authority (EFSA) proposed a daily intake limit being 4 g/kg body weight per day [30]. In 2018, the EU lowered the migration limit for BPA from 0.6 mg/kg to 0.05 mg/kg in food packaging and banned its use in baby food and infant formula [29]. The EU products containing BPA must now be labelled toxic for reproduction. The use of BPA in thermal paper has also been restricted since 2020 by EU regulations and REACH (Registration, Evaluation, Authorisation and Restriction of Chemicals) [27,29,31]. As a result, the industry has developed and gradually substituted BPA with more than 200 chemical analogues that are supposed to be safer alternatives [25,32,33,34].

Despite the increased production and use of BP analogues, little is known about whether the presence, chemical structures, and exposure risks of BPs are related to their effects. However, BPA has attracted considerable attention over the past two decades because of its toxicity [7,33,35]. Nevertheless, several BPA analogues have been detected in the environment, such as indoor dust, sediment, river water, soil, seawater, and sewage sludge, occasionally at concentrations similar to or greater than BPA [36]. Bisphenol-AP [4,4′-(1-phenylethylidene)bisphenol (BPAP)] is widely used for the synthesis of polyester polymers such as epoxy resins, polycarbonates, polyesters, epoxy resins, polyurethanes, polyethers, polyesters, polyacrylates, and polyether-polyacrylates [37,38], especially as indispensable plasticizers and flame retardants [39]. Although BPAP is a poorly studied BPA analogue, it has been confirmed as an endocrine-disrupting compound (EDC) [40]. Bisphenol C [4,4′-isopropylidenedi-o-cresol (BPC)] is one of the most versatile BPs used in a wide variety of products and articles such as flooring, curtains, furniture, paper products, construction materials, textiles, toys, electronic devices, and food packaging and storage [41]. ECHA evaluates BPC under the Community Rolling Action Plan (CoRAP) for suspected reproductive toxicity and possible endocrine disruption [29]. However, the structural similarity and physicochemical properties with BPA make the safety of these analogues questionable, especially since a growing number of studies show that some of them have similar or even higher toxic potential in terms of endocrine disrupting activity and reproductive toxicity than BPA [17,36,42,43,44]. They also have adverse genotoxic effects by inducing DNA strand breaks, impairing cell proliferation and cell cycle, altering the expression of genes involved in DNA damage response and repair, and causing many other changes in cell function [17,36,42,44,45].

A number of human biomonitoring studies have shown that the general population, including children, is often exposed to chemical mixtures present in the environment, food, and consumer products, rather than to a single compound [9]. Furthermore, exposure to bisphenols such as BPA rarely occurs in a single compound but rather through co-exposure to multiple bisphenols [46]. While the health effects of individual chemicals have been studied in detail, there are growing concerns about the potential health effects of co-exposure to multiple chemicals. However, regulatory requirements for mixtures are scarce, except for intentional mixtures such as formulated products and pharmaceuticals [9]. In addition, exposure to several chemicals can have combined effects, including additive or synergistic and ever-potentiating health effects. Given that various BP analogues coexist with BPA, it is important to understand the mechanisms and consequences of co-exposure to multiple analogues, preferably at environmentally relevant concentrations. Therefore, more research is needed to fully understand the health effects of co-exposure to bisphenols and to identify ways to mitigate these exposures.

**Figure 1:**
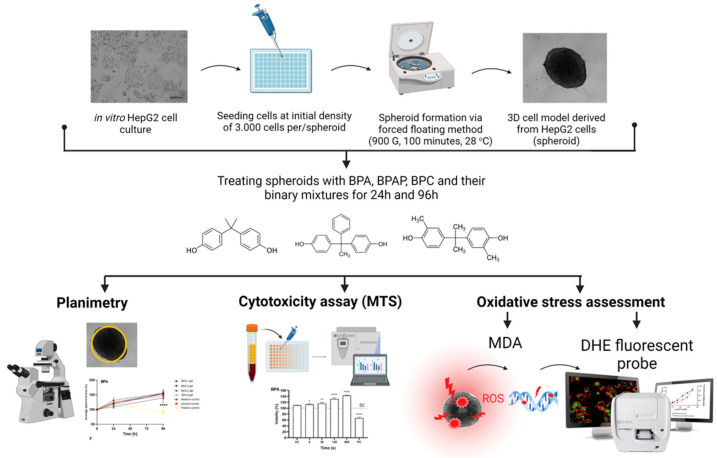
Schematic overview of the research process. Spheroids were formed from HepG2 single-cell suspension with an initial density of 3000 cells/spheroid using the forced floating method. After three days of culturing, spheroids were exposed to BPA, BPAP, BPC and their binary mixtures for 24 and 96 h. Various end-points were then measured, including planimetry, cell viability, and oxidative stress induction. The figure was made in BioRender program.

The present study investigated the potential cytotoxicity and induction of oxidative stress caused by BPA, BPAP, and BPC as single compounds or binary BPA/BPAP and BPA/BPC mixtures. BPAP and BPC were selected based on their occurrence in foodstuffs [36,47], human samples [48], and the structural diversity of their chemical formula. Their adverse effects were studied in in vitro 3D cell model (also called spheroids) formed from human hepatocellular carcinoma (HepG2) cells. In recent years, 3D cell models have gained popularity as preclinical test systems due to their enhanced metabolic, structural, and physiological properties compared to traditional 2D cell models, which do not adequately mimic the natural cell microenvironment represented by surrounding extracellular matrix and nearby cells [49,50,51,52,53,54,55,56]. The advantages of 3D cell models include their ability to grow undisturbed over longer periods, allowing for prolonged exposure to compounds, and making them suitable for chronic repeated dose studies [53,54,57,58]. They can also provide more predictive data for human exposure compared to classically cultured immortal hepatic cell lines [59,60]. Here, the spheroids were exposed to single bisphenols (BPA, BPAP and BPC) and binary combinations of BPA/BPAP and BPA/BPC for 24 and 96 h. The impact on spheroid growth, cell viability, and oxidative stress was evaluated (Figure 1).

## 2. Results and Discussion

BPA has become a significant concern worldwide due to its harmful effects on human health. These potential health effects have led to the increasing use of alternative bisphenols such as BPAP, BPC and many others. Due to the expected increase in the use of BPA analogues, exposure will also increase, and human co-exposure to these substances is inevitable. Unfortunately, not much is known about their toxicity, and there is a lack of information about their adverse effects, especially chronic exposure, and the combined effects of BPs. Therefore, this should be a priority for further investigation as there is insufficient information in the literature on co-exposure of BPs concerning their toxicity and mechanism of action. Human health risk assessment is still performed on data of individual compounds [31,61] where co-exposure to BPA with other BPs is not considered in the risk assessment. However, co-exposures to multiple BPs should not be neglected. Interactions between chemicals can occur, leading to additive, synergistic, or potentiating effects that may have more severe adverse health effects than individual compounds.

Therefore, this study investigated the effects of BPA, BPAP, and BPC as single compounds and their complex mixtures on metabolically competent HepG2 spheroids (See Table 1). The study evaluated the impact of these BPs on growth, average surface area, cell viability, and oxidative stress production. To obtain more relevant information on human exposure, we utilized in vitro 3D cell models, which are currently gaining importance in toxicological studies [51,52,55]. These models offer several advantages over traditional monolayer cell models, such as a higher level of liver-specific functions, including the activities of metabolic enzymes [55], direct cell-cell and cell-extracellular matrix interactions [59,62,63], and the cell morphology as well as their biochemical properties, more closely resemble the in vivo microenvironment. They also allow long-term, repeated dose studies that better reflect real human exposure scenarios [64]. To our knowledge, this is the first study to examine the combined effects of selected BPs in 3D cell models.

**Figure 2:**
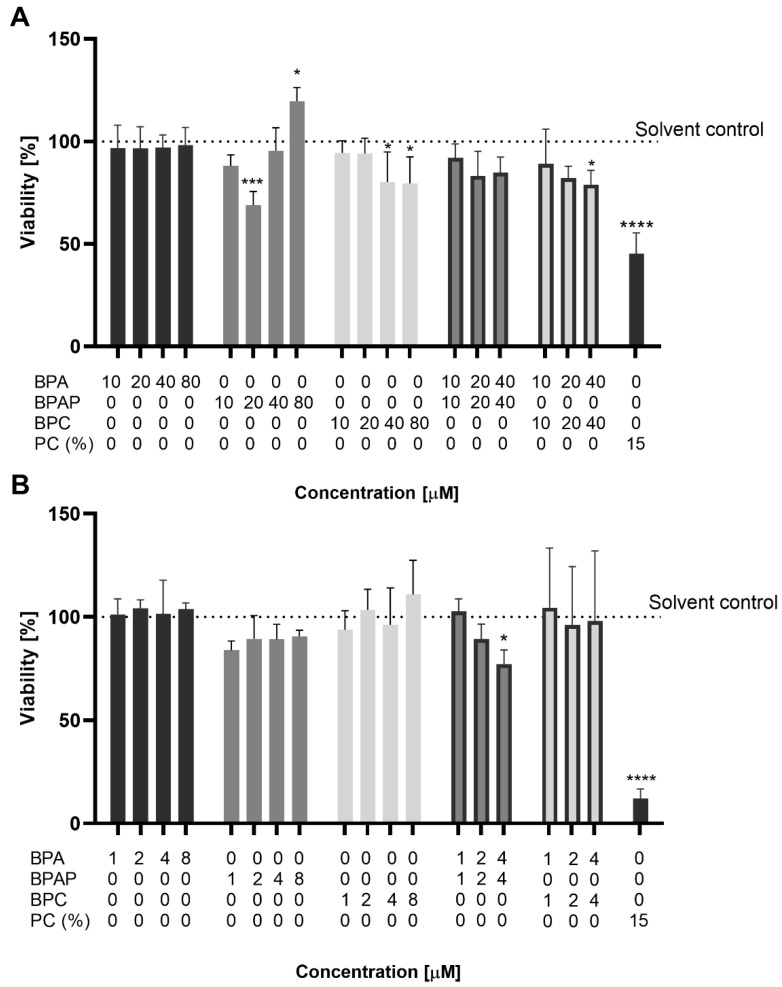
Viability of cells in HepG2 spheroids (MTS assay) after 24 h (**A**) and 96 h (**B**) exposure to BPA, BPAP, BPC and their binary mixtures. PC—positive control (15% DMSO). * significantly different from solvent control, * *p* < 0.05; *** *p* < 0.001; **** *p* > 0.0001 (one-way ANOVA; Dunnett’s multiple comparison test).

The effect of BPA, BPAP, BPC, and their combinations on the viability of cells in 3-day-old HepG2 spheroids was evaluated using the MTS assay after short-term (24 h) and long-term exposure (96 h) (for concentrations, see Table 2). The results showed that BPA at applied concentrations (up to 80 µM and 8 µM, respectively) did not affect cell viability after 24- or 96-h exposure (Figure 2). On the contrary, BPAP and BPC affected cell viability after 24-h exposure (Figure 2A). BPAP at 20 µM significantly reduced cell viability by 32 ± 6.7% compared to the solvent control, while no reduction in cell viability was observed at higher concentrations. At 80 µM, mitochondrial dehydrogenase enzyme activity increased significantly by 19.6 ± 6.7%, suggesting stress-induced changes in cells as a response to the harmful effects of BPAP. BPC significantly reduced cell viability in a dose-dependent manner at ≥40 μM (Figure 2A). Similar findings for BPAP were reported by Sendra et al. [20], who observed an increase in cell viability of HepG2 spheroids after 24-h exposure to 20 µM BPAP. In the same study BPC at ≥20 μM reduced cell viability after 24-h exposure, but the reduction was not statistically significant. Previously BPA was reported to increase cell viability in HepG2 monolayer culture (1 and 10 µM) [65], suggesting that the observed increase of mitochondrial dehydrogenase enzyme activity is a sign of toxicity. In monolayer HepG2 cell cultures, BPA did not affect cell viability at concentrations up to 80 µM after 24 h exposure [17,44]. At concentrations greater than 200 µM, Ozyurt et al. [66], reported that BPA can cause a concentration-dependent decrease in HepG2 cell viability, while Yu et al. [67] reported cytotoxicity at concentrations as low as 10 µM. Several studies reported that BPA analogues could have higher cytotoxic potential in mammalian cell lines than BPA [44,68,69,70]. Padberg et al. showed that BPC is more toxic than BPA in the HepG2 cell line [71].

There is limited information on the cytotoxicity of co-exposure to BPA and its analogues used in industry. Therefore, in our study, we investigated the effect of binary mixtures, namely a combination of BPA and BPAP and a combination of BPA and BPC, on HepG2 spheroids at concentrations 10 + 10, 20 + 20, 40 + 40 µM and 1 + 1, 2 + 2, 4 + 4 µM after 24 and 96 h of exposure, respectively (Figure 2A,B). Both mixtures at the highest concentrations tested decreased cell viability. The BPA/BPAP combination at a concentration of 40 + 40 µM decreased cell viability by 15 ± 7.1% after 24 h, while after 96 h of exposure, cell viability was statistically significantly decreased by 23 ± 6.9%, even at 10-fold lower concentrations. Similarly, the BPA/BPC combination at 40 + 40 µM decreased cell viability by 22 ± 7.1% after 24 h of exposure but had no effect after 96 h. The binary mixtures decreased HepG2 cell viability to a similar extent as the single compounds, except for the BPA/BPAP combination at 4 + 4 μM after 96 h exposure. Both BPs at 4 and 8 μM did not affect cell viability, whereas the combination statistically significantly decreased cell viability by 23%. Similarly, Skledar and Mašič [72] observed no cytotoxic effect of BPA and BPC and their combinations at concentrations up to 25 µM on the ERα-Hela 9903 cell line.

There are almost no publications available in the literature addressing mechanisms of toxicity due to co-exposure to multiple BP analogues or BP analogues together with other environmental toxins. Most of the available toxicity studies have evaluated the effects of a single BP [17,20,44]. However, it is extremely important to consider potential additive or synergistic effects caused by a mixture of BPs analogues when studying the mechanisms of action and assessing human health risks.

**Figure 3:**
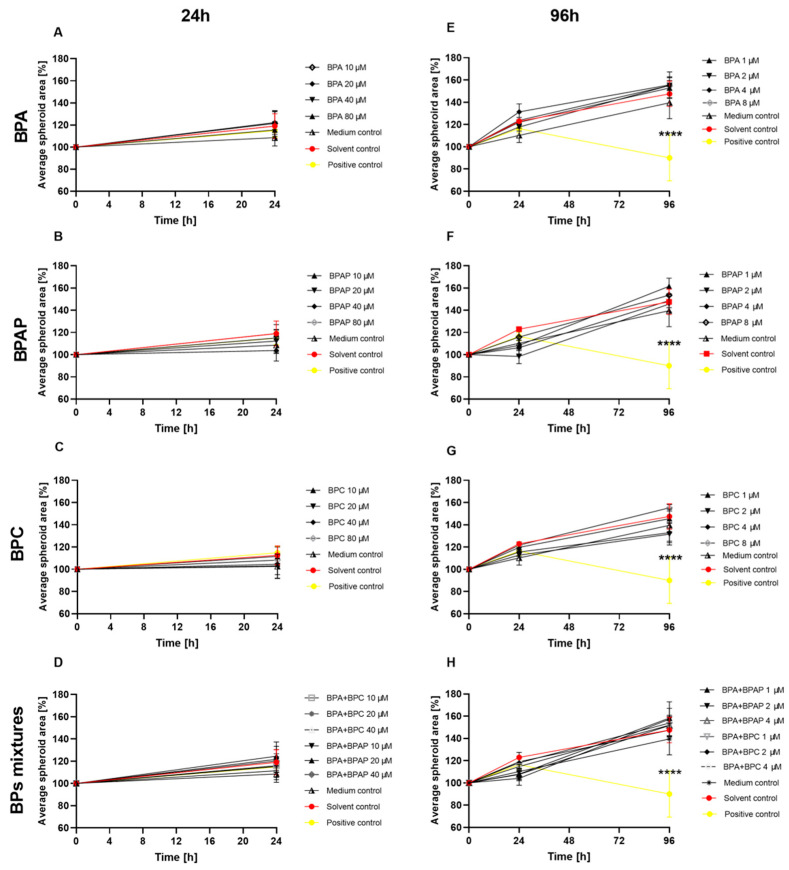
Planimetry measurements of spheroids (3-day old) before treatment (0) and after 24 and 96-h treatment with BPA, BPAP, BPC (**A**–**C**,**E**–**G**) and their binary mixtures (**D**,**H**). (**A**–**D**) Figures show the change in % of the average spheroid area after 24 h of exposure compared to the average area at the beginning of treatment. (**E**–**H**) Figures show the change in % of the average spheroid area after 24 h and 96 h of exposure compared to the average area of the 72-h old spheroid. The growth of spheroids was monitored at 4× magnification. 15% DMSO served as a positive control (PC). Results are presented as mean ± SD (N = 10) of three biologically independent experiments. * different from solvent control, **** *p* < 0.0001 (one-way ANOVA; Dunnett’s multiple comparison test).

We further investigated the impact of BPA, BPAP and BPC and their binary mixtures on the growth, size, and average surface area of HepG2 spheroids over 24 (Figure 3A–D) and 96 (Figure 3E–H) hours of exposure. Using a planimetry approach, we found that the BPs and their binary mixtures at the concentrations applied did not affect the size and average surface area of HepG2 spheroids up to 96 h of exposure compared to solvent control. The BP-exposed spheroids grew to a similar extent as spheroids exposed to the solvent control (Figure 3 and Tables S1 and S2). The positive control, 15% DMSO, decreased the average surface area by 10 ± 20.7% after 96 h of exposure, compared to the starting average surface area (set as 100%). In addition, light microscopy analysis revealed no changes in the roundness, size, shape, or compactness of the spheroids after 24 and 96 h of exposure to BPs and their binary mixtures compared to the solvent control, except for the positive control after 96 h of exposure (Figures S1 and S2). To date, only a few studies have investigated the effects of BPA and its analogues on growth, compactness, and surface area, and these have been conducted on traditional 2D monolayer cultures [73,74,75]. The results of the only published study in a 3D HepG2 cell model [20] show no significant changes in the average surface area after 24 h of exposure to single BPA, BPAP, and BPC compounds compared to the average surface area of the solvent control. In addition, concentrations up to 80 µM of BPs studied did not affect the average surface area of spheroids after prolonged exposure (96 h).

Oxidative stress is a common condition believed to majorly impact cancer development [76,77]. Normally, cells produce a small amount of reactive oxygen species (ROS), which are beneficial for their effects on the redox state of cells and play an important role in the signalling cascade [78]. However, excess ROS can cause oxidative stress and damage lipids, proteins, and cell membranes, leading to structural and functional disruption of cells [79]. Several literature data link BPA to elevated oxidative stress [66,80,81], and some studies have shown that BPs analogues such as BPF, BPS, BPAF, and BPB can induce oxidative stress in liver cells and other cell lines [42,66,82,83,84,85,86,87]. However, no data are available for BPAP and BPAC and combined effects with BPA. In this study, we used for the first time HepG2 spheroids to investigate the induction of oxidative stress by the BPs studied. In addition, the thiobarbituric acid assay (TBA) was used to measure the content of malondialdehyde (MDA), a biomarker of lipid peroxidation that reflects the extent of oxidative damage to cells [88,89,90], and the dihydroethidium (DHE) fluorescent probe was used to evaluate changes in intracellular ROS levels after exposure to BPA, BPAP, BPC, and their binary mixtures.

**Figure 4:**
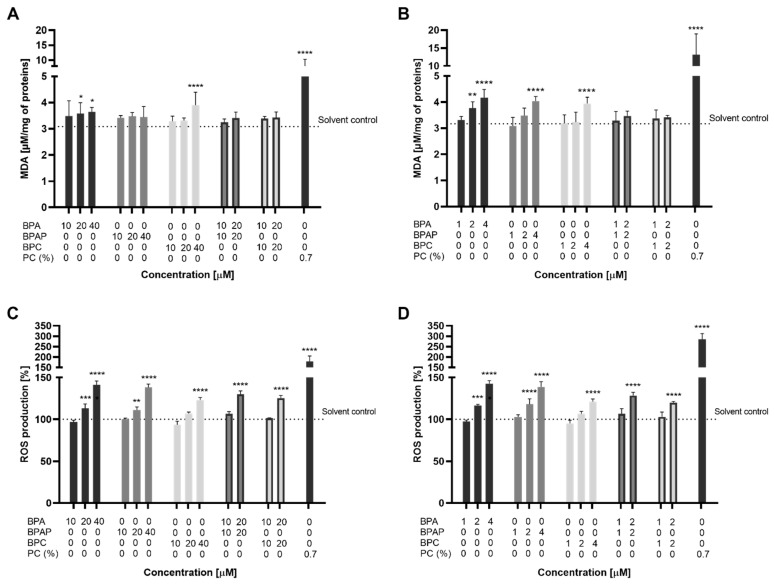
Determination of oxidative stress by TBA assay (MDA) (**A**,**B**) and DHE fluorescent probe (ROS production) (**C**,**D**) after exposure to BPA, BPAP, BPC and their binary mixtures for 24 h (**A**,**C**) and 96 h (**B**,**D**). PC—positive control (Luperox^®^ TBH70X (0.7 µM). * different from solvent control, * *p* < 0.05; ** *p* < 0.01; *** *p* < 0.001; **** *p* < 0.0001 (One-way ANOVA; Dunnett’s multiple comparison test).

The results showed that MDA levels were significantly elevated in spheroids exposed to BPA for 24 h at 20 (3.57 ± 0.42 µM/mg of protein) and 40 µM (3.64 ± 0.17 µM/mg of protein), compared to the solvent control (3.08 ± 0.20 µM/mg of protein) (Figure 4A). Increased MDA levels after 24-h exposure were observed in spheroids exposed to 40 µM BPC (3.90 ± 0.49 µM/mg of protein) but not for BPAP and binary mixtures at applied conditions. After 96 h of exposure of spheroids to BPA, the content of MDA was significantly elevated at 2 (3.76 ± 0.24 µM/mg of protein) and 4 µM (4.17 ± 0.31 µM/mg of protein), compared to the solvent control (3.17 ± 0.08 µM/mg of protein) (Figure 4B). A significant increase of MDA was also induced by BPAP at 4 µM (4.04 ± 0.18 µM/mg of protein) and BPC at 4 µM (3.94 ± 0.24 µM/mg of protein). On the contrary, binary mixtures (BPA/BPAP and BPA/BPC) did not have a statistically significant effect on MDA levels compared to the solvent control, which suggests an antagonistic effect. Positive control (0.7 μM Luperox^®^ TBH70X) significantly induce the formation of MDA by approximately 2.3 (8.02 ± 2.07 µM/mg of protein) and 3.97-fold change (13.12 ± 5.3) µM/mg of protein) after 24 and 96 h of exposure, respectively, compared to solvent control. In contrast to our results, Ozyurt et al. [66] showed that BPA at 397 µM (IC_30_ concentration) did not affect MDA levels in HepG2 monolayers after 24 h of exposure. However, they showed that a high dose of BPA increased free radical formation and decreased the ability to detoxify ROS.

An increase in intracellular ROS causes oxidative stress and damage to organelles and cellular macromolecules [66]. The present study assessed changes in intracellular ROS levels following exposure to BPA, BPAP, BPC, and their binary mixtures using the fluorescent dihydroethidium (DHE) probe. The results showed that all three bisphenols significantly increased the intracellular ROS levels in HepG2 spheroids in a dose-dependent manner after short and prolonged exposure times (Figure 4C,D). BPA induced a significant increase in ROS production at 20 and 40 µM by 13 ± 5.27% and 41 ± 4.58% compared to solvent control (set at 100%) after 24 h of exposure (Figure 4C). Similarly, also BPAP at 20 and 40 µM resulted in 11 ± 3.57% and 38 ± 3.90%, while BPC was the least potent and induced a significant increase in ROS formation only at the highest concentration tested at 40 µM by 23 ± 2.97%. The induction of ROS formation was also observed at the highest tested concentration of both binary mixtures (20 + 20 µM), BPA/BPAP (by 29 ± 2.43%) and BPA/BPC (by 25 ± 3.31) again being less potent. After 96 h of exposure, BPA increased ROS formation at 2 and 4 µM by 16 ± 1.39% and 42 ± 3.51%, respectively, compared to the solvent control (Figure 4D). Similarly, BPAP elevated ROS production at 2 and 4 µM by 18 ± 6.36% and 38 ± 6.38%. The lowest induction of ROS was caused by BPC, only at 4 µM by 21 ± 3.05%. The binary mixtures (2 + 2 µM) of BPA/BPAP and BPA/BPC increased ROS levels by 28 ± 3.05% and 20 ± 1.11%, respectively, where the combination BPA/BPC was less potent than BPA/BPAP (Figure 4C,D). Positive control significantly induced ROS formation by 78 ± 21.4% and 184 ± 22.8% after 24 and 96 h of exposure, respectively, compared to the solvent control.

Overall, these results indicate that all investigated bisphenols and their binary mixtures caused oxidative stress in HepG2 spheroids, with BPC being the least potent, while BPA and BPAP induced oxidative stress to a similar extent. The findings align with previous studies on HepG2 monolayer cultures, which showed that a high dose of BPA (397 µM) and other analogues could increase free radical formation after 24 h of exposure [66]. In addition, BPA has been shown to induce MDA content and ROS formation in several cell types in vitro at concentrations ranging from nanomolar to micromolar [64,87,91,92,93,94,95,96,97]. Similarly, ROS induction has been reported for several BPA analogues, including BPS, BPF and BPAF in human peripheral blood mononuclear cells [83,87] and human erythrocytes [71]. However, for BPAP and BPC, particularly their mixtures, limited data are available on the induction of oxidative stress. BPAP and BPC have been described to induce ROS formation in IMR-32 cells [98] and BPC also in MCF-7 cells [99].

The findings suggest that exposure to BPA, BPAP, BPC, and their mixtures can increase cellular oxidative stress. In addition, the increased formation of ROS can lead to the induction of DNA damage. Previously, it was reported that all three bisphenols studied induced increased formation of DNA single-strand breaks in HepG2 spheroids as determined by the comet assay, with BPAP being the most effective [20]. To fully understand the mechanisms and the potential health risks associated with exposure to these chemicals, further research is needed.

## 3. Materials and Methods

### 3.1. Chemicals and Preparation of Bisphenol Standard Solution

Bisphenol A (4,4’-(propane-2,2-diyl)diphenol; BPA), Bisphenol AP (4,4′-(1-Phenylethylidene)bisphenol; BPAP), Bisphenol C (4,4’-(2,2-dichloroethene-1,1-diyl)diphenol; BPC), Penicillin/streptomycin, Na-pyruvate, L-glutamine, Dimethylsulphoxide (DMSO), Bovine Serum Albumin (BSA), Coomassie Brilliant Blue G-259, Thiobarbituric acid (TBA), Dihydroethidium (DHE), and Methylcellulose were obtained from Sigma-Aldrich (St. Louis, MO, USA). Minimum essential medium eagle (MEME-10370-047), Trypsin-EDTA (0.25%), TripLE Express (12604-013), Foetal bovine serum (FBS) and Trypan Blue (15250-061) were obtained from Gibco (Praisley, Scotland, UK). Etoposide (ET) was from Santa Cruz Biotechnology (St. Cruz, CA, USA). Phosphate buffered saline (PBS) was purchased from PAA Laboratories (Dartmouth, NH, USA). Collagenase (Type I- 17018029) was obtained from Fisher Sciences (Branchburg, NJ, USA). CellTiter 96^®^ AQueous cell proliferation assay (3-(4,5-dimethylthiazol-2-yl)-2,5-diphenyltetrazolium bromide; MTS) was obtained from Promega (Madison, WI, USA). Phosphoric acid (H_3_PO_4_) was from Merck (Darmstadt, Germany). Luperox^®^ TBH70X (0.7 mM)- tert-Butyl hydroperoxide was obtained from Aldrich Chemistry (USA).

Standard stock solutions of BPA (16 mM), BPAP (16 mM) and BPC (16 mM) were prepared in DMSO and stored at −20 °C, and dilutions for cell treatment were prepared in cell medium each time fresh before treatment. The final concentration of DMSO in BPs solutions for cell treatment did not exceed 0.3%. In treatment concentrations, the concentration of DMSO was adjusted to 0.3% and 0.06% for 24 h and 96 h exposures, respectively.

**Table 1:** UPAC name, CAS N°, structural formulas, the molecular mass of BPA, and its analogues BPAP and BPC.

Compound Name	IUPAC Name	CAS No	Structural Formula	Molecular Weight [g/mol]
Bisphenol A	2,2-bis(4-hydroxyphenyl)propane	80-05-07	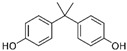	228.29
Bisphenol AP	4,4′-(1-phenylethylidene)bisphenol	1571-75-1	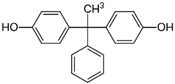	290.36
Bisphenol C	4,4′-Isopropylidenedi-o-cresol	79-97-0	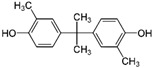	256.34

### 3.2. Cell Culture and Formation of In Vitro 3D Cell Model

The human hepatocellular carcinoma (HepG2) cell line was obtained from the ATCC cell bank (ATCC-HB-8065™, Manassas, VA, USA). Cells were grown in MEME medium containing NEAA supplemented with 10% FBS, 2.2 g/L NaHCO3, 2 mM L-glutamine, 100 IU/mL penicillin/streptomycin, and 1 mM sodium pyruvate at 37 °C in a humidified atmosphere with 5% CO_2_. For the experiments, 3D cell models (spheroids) were formed from single-cell suspension using the forced floating method described by Štampar et al. (2019) [56]. Spheroids with an initial cell density of 3000 cells per spheroid were seeded onto 96-well plates and were grown for three days before treatment.

### 3.3. Preparation of Complex BPs Mixtures and Treatment Conditions

After three days of culture, the growth medium was removed, and the spheroids were exposed to the following bisphenols: BPA, BPAP, and BPC as single compounds (BPs; Table 1) and their binary mixtures in a 1:1 ratio (BPA + BPAP; BPA + BPC) for 24 h and 96 h. For the 96-h exposure, the medium was replaced after 48 h of treatment with a fresh medium containing the same concentration of BPs (Table 2). A solvent control (SC) and appropriate positive controls (PC) were used in all experiments.

**Table 2:** Treatment conditions and selected concentration for the experiments.

Time of Exposure	Single Compound or Binary Mixture	Concentrations
24 h	BPA, BPAP, BPC	10 µM, 20 µM, 40 µM, 80 µM
	BPA + BPAP/BPC	10 + 10 µM, 20 + 20 µM, 40 + 40 µM
96 h	BPA, BPAP, BPC	1 µM, 2 µM, 4 µM, 8 µM
	BPA + BPAP/BPC	1 + 1 µM, 2 + 2 µM, 4 + 4 µM

### 3.4. Cytotoxicity—The MTS Assay

The cytotoxicity of BPA, BPC, BPAP and their binary mixture was assessed using the tetrazolium-based (MTS) assay according to the manufacturer’s instructions (Promega) with minor modifications [44,56]. Three-day-old spheroids were exposed to single compounds BPA, BPC, and BPAP at concentrations of 10 µM, 20 µM, 40 µM, and 80 µM and 1 µM, 2 µM, 4 µM, and 8 µM for 24 and 96 h, respectively, and their combinations at concentrations of 10 + 10 µM, 20 + 20 µM, and 40 + 40 µM and 1 + 1 µM, 2 + 2 µM, and 4 + 4 µM for 24 and 96 h, respectively (see Table 2). At the end of the treatment period (after 24 or 96 h), 20% of a freshly prepared mixture of MTS: PMS solution (20:1) was added to each well and incubated for 3 h. After incubation, absorbance was measured at 490 nm using a microplate reader (Sinergy MX, BioTek, Winooski, VT, USA). The experiment was performed with five replicates per treatment point and repeated in three independent biological replicates. Each experiment included a negative control (cell medium), a solvent control (cells exposed to 0.3% and 0.06% DMSO for 24 and 96 h, respectively), and a positive control (15% DMSO).

Significant differences in cell viability between the treated groups and the solvent control group were analyzed using a One-Way Analysis of Variance (ANOVA) and Dunnett’s multiple comparison test using GraphPad Prism V8 (GraphPad Software, San Diego, CA, USA). [*p* < 0.05 (*), *p* < 0.001 (**), *p* < 0.0001 (***) were considered statistically significant].

### 3.5. Planimetry—The Effects on the Average Surface Area

Surface area measurements (µm^2^) and micrographs of at least five spheroids per treatment point were recorded from day three (immediately before the BP treatment = time 0) and after 24 and 96 h of post-treatment. Spheroids were exposed to single compounds at concentrations 10, 20, 40, and 80 µM and 1, 2, 4, and 8 µM for 24 and 96 h, respectively, and their combinations at concentrations of 10 + 10 µM, 20 + 20 µM, and 40 + 40 µM and 1 + 1 µM, 2 + 2 µM, and 4 + 4 µM for 24 and 96 h, respectively (see Table 2). Average surface area measurements and micrographs were captured using Cytation 5 (BioTek, Winooski, VT, USA) with a built-in microscope and a wide-angle-view camera at 4× magnification, and analysis was performed using the Gen5 program (Software for imaging and microscopy, BioTek, USA, version 3.11). Experiments were repeated three times in independent biological replicates.

In each experiment, a negative control (cell medium), solvent control (cells exposed to 0.3% and 0.06% DMSO for 24 and 96 h, respectively), and positive control (15% DMSO) were included. Graphical and statistical analyses were performed using GraphPad 8 software by one-way analysis of variance (ANOVA) test with a Dunnett post hoc [*p* < 0.05 (*), *p* < 0.001 (**), *p* < 0.0001 (***) were considered statistically significant].

### 3.6. Oxidative Stress—The MDA Assay and ROS Production

Three-day-old spheroids were exposed to single compounds BPA, BPC, and BPAP at concentrations 10, 20 and 40 µM and 1, 2 and 4 µM for 24 and 96 h, respectively, and their binary mixtures at concentrations of 10 + 10 µM and 20 + 20 µM, and 1 + 1 µM and 2 + 2 µM for 24 and 96 h, respectively (see Table 2). Before measuring oxidative stress parameters, 24 spheroids were collected for each sample in a 5 mL Eppendorf tube. After collection, the spheroids were centrifuged at 188 g for 4 min, washed with 1 mL PBS and stored in 500 µL PBS at −80 °C. Protein content, malondialdehyde (MDA), and reactive oxygen species (ROS) production were determined in the collected samples. The experiments were performed in three independent biological replicates. Each experiment included a negative control (cell medium), a solvent control (cells exposed to 0.3% and 0.06% DMSO for 24 and 96 h, respectively), and a positive control (Luperox^®^ TBH70X (0.7 µM)).

#### 3.6.1. Determination of MDA Level

Malondialdehyde (MDA) is one of the end products of the peroxidation of polyunsaturated fatty acids in cells. An increase in free radicals leads to an overproduction of MDA. Measurement of the MDA level in the collected samples was performed using the TBA assay previously described by Domijan et al. [90] with minor modifications. First, a reagent (0.6% TBA in 1% H_3_PO_4_) was prepared. Spheroids stored in PBS were homogenized in ice-cold conditions and then centrifuged at 10,000× *g* for 8 min. 50 µL of the supernatant was added to 100 µL of the reagent. The samples were mixed and incubated for 30 min in a thermostatic block at 90 °C. To stop the reaction, the samples were placed on ice. 100 µL of the reaction mixture was transferred to 96-well microtiter plates, and absorbance was read at 532 nm (SpectraMax i3x, Molecular Devices, San Jose, CA, USA). The MDA concentration was calculated based on the Beer-Lambert law and the absorption coefficient of MDA (156 mM^−1^ cm^−1^). As the number of cells in the spheroids can vary, affecting the MDA level, the MDA concentration was normalized based on the protein level in the same sample. The protein level in the supernatant was determined according to Bradford [100]. The absorbance of the reaction mixture was read on a microplate reader (SpectraMax i3x, Molecular Devices, San Jose, CA, USA) at 595 nm. The protein concentration was quantified using a calibration curve prepared from BSA standards (Table S3).

Significant differences between the treated and solvent control groups were analysed by One-way analysis of variance (ANOVA) and Dunnett’s multiple comparison test using GraphPad Prism V8 (GraphPad Software, San Diego, CA, USA). [*p* < 0.05 (*), *p* < 0.001 (**), *p* < 0.0001 (***) were considered statistically significant].

#### 3.6.2. Determination of ROS Production

The level of intracellular ROS production in HepG2 spheroids after BP treatment was determined using fluorescent probe DHE as described in Gajski et al. [101]. DHE is a membrane-permeable compound oxidized mainly by the superoxide radical (O_2-_) to the red fluorescent ethidium (DNA-binding membrane-impermeable compound). Hence, an increase in red fluorescence indicates an increase in the superoxide radical. Therefore, 50 µL of the previously obtained sample supernatant was transferred to a black 96-well microtiter plate, followed by the addition of 50 µL of DHE (10 µM). Fluorescence intensity was measured using a microplate reader set at λ_ex_ 535 nm and λ_em_ 635 nm (SpectraMax i3x, Molecular Devices, San Jose, CA, USA). In addition, 50 µL of deionized H_2_O was used as a reference measurement (blank), which was treated the same way as the samples.

Significant differences between the treated and solvent control groups were analysed by one-way analysis of variance (ANOVA) and Dunnett’s multiple comparison test using GraphPad Prism V8 (GraphPad Software, San Diego, CA, USA). [*p* < 0.05 (*), *p* < 0.001 (**), *p* < 0.0001 (***) were considered statistically significant].

## 4. Conclusions

The present study investigated the toxic effects of BPA, BPAP, BPC, and binary mixtures of BPA/BPAP and BPA/BPC in an in vitro 3D HepG2 cell model. The results showed that BPA did not affect cell viability under the applied conditions. In contrast, after short-term exposure, BPAP and BPC and the binary mixtures (BPA/BPAP and BPA/BPC) decreased cell viability in the HepG2 3D cell model. None of the bisphenols studied affected cell viability after prolonged exposure, except for the BPA/BPAP binary mixture. In addition, none of the bisphenols and their binary mixtures studied affected spheroids’ compactness, size, surface area, and growth. All three bisphenols and their binary mixtures induced increased MDA and ROS levels, with BPC being the least effective. Overall, the results of the present study indicate that BPA and its analogues, BPAP and BPC, induce oxidative stress, which is involved in their toxic activity. However, further research is needed to fully understand the mechanisms and potential health risks associated with exposure to the studied bisphenols. It is important to investigate the effects of complex mixtures, as the combined effects may differ from those induced by single compounds.

## Data Availability

The authors confirm that the data supporting the findings of this study are available within the article [and/or] its supplementary materials. Generated raw data of this study are available from the corresponding author [B.Ž.] on request.

## Supplementary Materials

The following supporting information can be downloaded at: https://www.mdpi.com/article/10.3390/molecules28073085/s1. Data showing the impact on the growth of spheroids, Figures showing the impact on the growth of spheroids and Data on protein concentration (mg/mL).

## Sample Availability

Samples of the compounds are not available from the authors.
