# Supplementary material for: Combined Toxic Effects of BPA and Its Two Analogues BPAP and BPC in a 3D HepG2 Cell Model
Source: Molecules. 2023 Mar 30;28(7):3085. doi: 10.3390/molecules28073085 (PMC10095618; doi:10.3390/molecules28073085)

SUPPORTING INFORMATION

SI-1 Results

Table S1. Measurements of average surface area of spheroids (µm^2) at time 0 (3-days old) and after 24-h exposure to BPs and their combinations. The measurement of a surface area were taken before treatment (time 0) and after 24 h of exposure at 4x magnification using Cytation 5 (BioTek, Winooski, VT, USA). 15 % DMSO served as a positive control (PC). The experiment was conducted at least in five replicates and in three biologically independent experiments.

		**Average surface area at time 0 (before treatment)**		**Average surface area after 24 h of treatment**	
**BPs**	**Concentration (µM)**	Average spheroid area (µm^2)	SD	Average spheroid area (µm^2)	SD
**SC**		230760	29788	281083	31484
**BPA**	10	226500	13457	286611	33389
	20	236167	12655	288500	30721
	40	226337	13923	281222	36975
	80	221485	12079	272944	20490
**BPAP**	10	228862	15091	280667	9134
	20	223089	8074	264778	28065
	40	213167	11856	245056	23277
	80	232694	35623	271444	32633
**BPC**	10	225667	29309	283522	24154
	20	230333	19043	261972	29212
	40	237825	24022	265667	25007
	80	222537	8406	275167	15940
**BPA+BPAP**	10+10	233824	19325	287222	33593
	20+20	254053	58763	293889	37801
	40+40	228347	7298	283722	29310
**BPA+BPC**	10+10	218933	32916	294667	31687
	20+20	224333	8565	292667	14449
	40+40	236500	29620	282944	23083
**PC**	15 % DMSO	229667	26157	305550	42956

Table S2. Measurements of average surface area of spheroids (µm2) at time 0 (3-days old) and after 24 and 96 h exposure to BPs and their combinations to 10x lower concentrations. The measurement of a surface area were taken before treatment (time 0) and after 24 and 96 h of exposure at 4x magnification using Cytation 5 (BioTek, Winooski, VT, USA). 15 % DMSO served as a positive control (PC). The experiment was conducted at least in five replicates and in three biologically independent experiments.

		**Average surface area at time 0 (before treatment)**		**Average surface area after 24 h of treatment**		**Average surface area after 96 h of treatment**	
**BPs**	**Concentration (µM)**	Average spheroid area (µm2)	SD	Average spheroid area (µm^2)	SD	Average spheroid area (µm^2)	SD
**SC**		228000	26885	265667	3055	385472	16393
**BPA**	1	233667	8116	260004	12166	388416	28952
	2	238500	12744	276333	18016	382875	30228
	4	221409	19416	262666	12345	389500	34648
	8	225720	23301	272333	18709	391222	28359
**BPAP**	1	197803	9605	258667	20848	385833	28191
	2	199322	14846	235000	17531	347722	24679
	4	225750	28324	253667	17021	355167	22503
	8	223568	16784	276667	16932	366500	2121
**BPC**	1	246533	14417	304000	14933	369750	35229
	2	266537	27744	286333	6658	334396	33005
	4	250667	33416	293667	12014	337625	30467
	8	281500	25727	309333	577	394667	11504
**BPA+BPAP**	1+1	207600	6831	257667	13051	377875	33057
	2+2	225400	7236	248667	15503	375500	58690
	4+4	237600	39841	257000	14442	362334	32999
**BPA+BPC**	1+1	226467	20696	291333	10017	386083	15464
	2+2	227893	10210	299667	17559	390778	22364
	4+4	241671	13626	302667	25580	374563	22776
**PC**	15 % DMSO	235550	12093	301333	20333	215600	53174

Figure S1. Representative light microscopy micrographs showing changes in average surface area, compactness, and shape of 72-h-old spheroids exposed to BPs for 24-h. The micrographs were taken before treatment (time 0) and after 24 h of exposure at 4x magnification using Cytation 5 (BioTek, Winooski, VT, USA). 15 % DMSO served as a positive control (PC). The experiment was conducted at least in five replicates and in three biologically independent experiments.

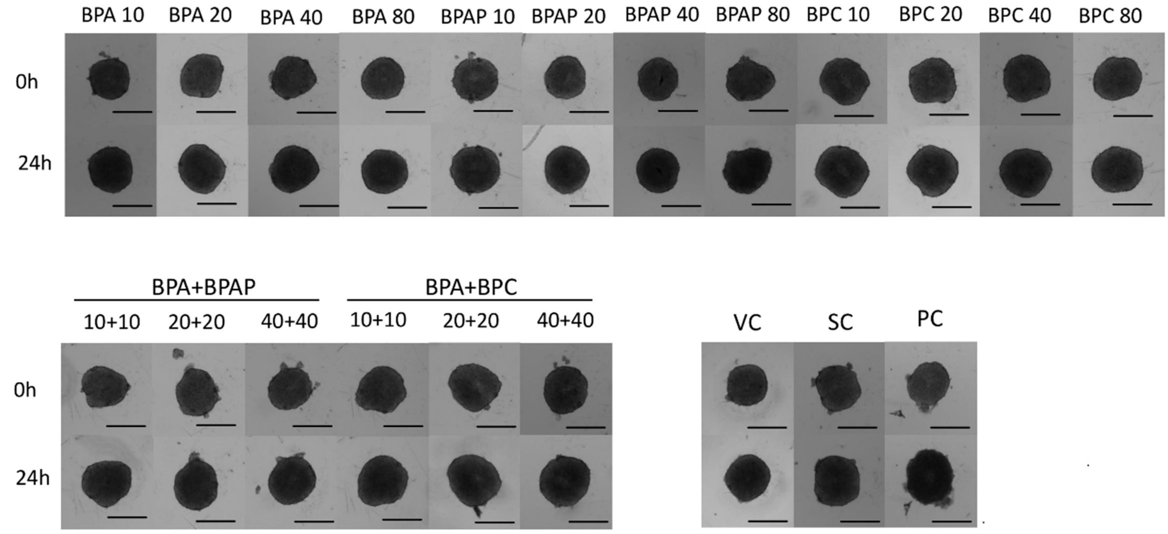

Figure S2. Representative light microscopy micrographs showing changes in average surface area, compactness, and shape of 72-h-old spheroids exposed to BPs for 24-h and 96h to 10x lower concentrations. The micrographs were taken before treatment (time 0) and after 24 and 96 h of exposure at 4x magnification using Cytation 5 (BioTek, Winooski, VT, USA). 15 % DMSO served as a positive control (PC). The experiment was conducted at least in five replicates and in three biologically independent experiments.

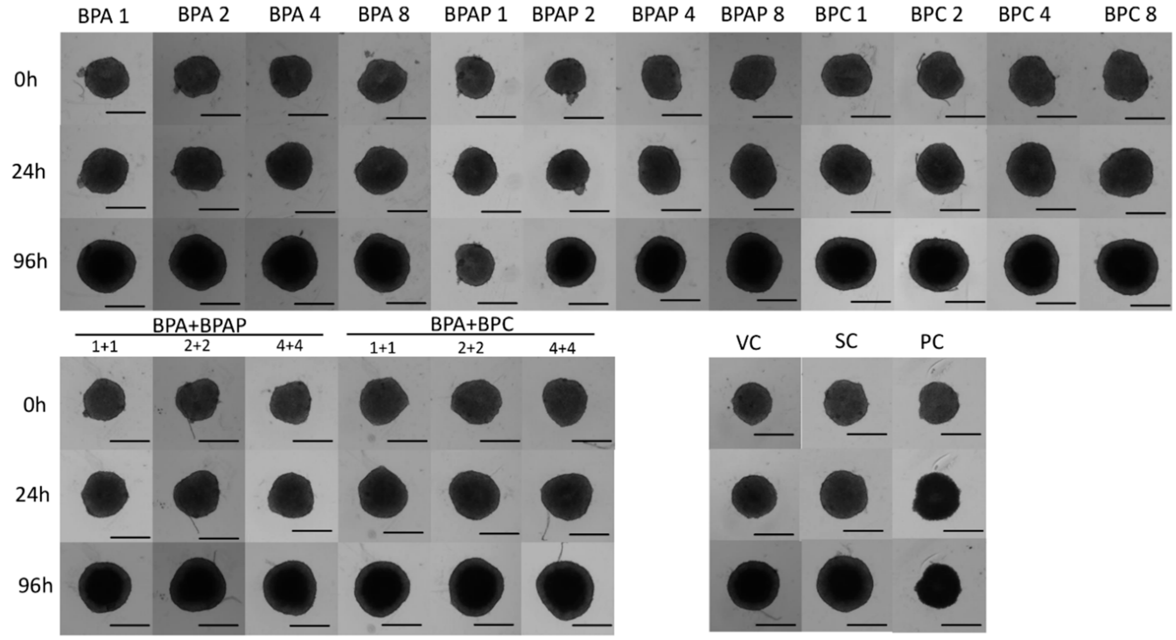

Table S3. Determination of protein concentration (mg/ml) in spheroids exposed to BPs and their binary mixtures for 24h and 96h by Bradford assay. PC - positive control (Luperox® TBH70X (0.7 μM). Measurements were done in three independent biological experiments.

**24 h**		**96 h**	
**Con.**	Protein concentration per sample [mg/ml]	**Con.**	Protein concentration per sample [mg/ml]
**μM**	**Ave. ± SD**	**μM**	**Ave. ± SD**
0	0,3633 ± 0,0062	0	0,4335 ± 0,0437
	**BPA**		**BPA**
10	0,3215 ± 0,0563	1	0,4481 ± 0,0407
20	0,3218 ± 0,0581	2	0,4261 ± 0,0493
40	0,3475 ± 0,0268	4	0,4442 ± 0,0532
	**BPAP**		**BPAP**
10	0,3115 ± 0,0223	1	0,4447 ± 0,0608
20	0,3120 ± 0,0249	2	0,4223 ± 0,0499
40	0,3015 ± 0,0269	4	0,4150 ± 0,0361
	**BPC**		**BPC**
10	0,3276 ± 0,0460	1	0,4372 ± 0,0568
20	0,3468 ± 0,0223	2	0,4484 ± 0,0636
40	0,3104 ± 0,0400	4	0,4182 ± 0,0332
	**BPA+BPAP**		**BPA+BPAP**
10+10	0,3349 ± 0,0211	1+1	0,4301 ± 0,0477
20+20	0,3287 ± 0,0266	2+2	0,4207 ± 0,0224
	**BPA+BPC**		**BPA+BPC**
10+10	0,3226 ± 0,0351	1+1	0,4339 ± 0,0362
20+20	0,3455 ± 0,0285	2+2	0,4551 ± 0,0060
	**PC**		**PC**
0,7	0,0632 ± 0,0220	0,7	0,0514 ± 0,0329

Figure S3. Standard curve for determination of protein content in samples, acquired by Bradford assay.

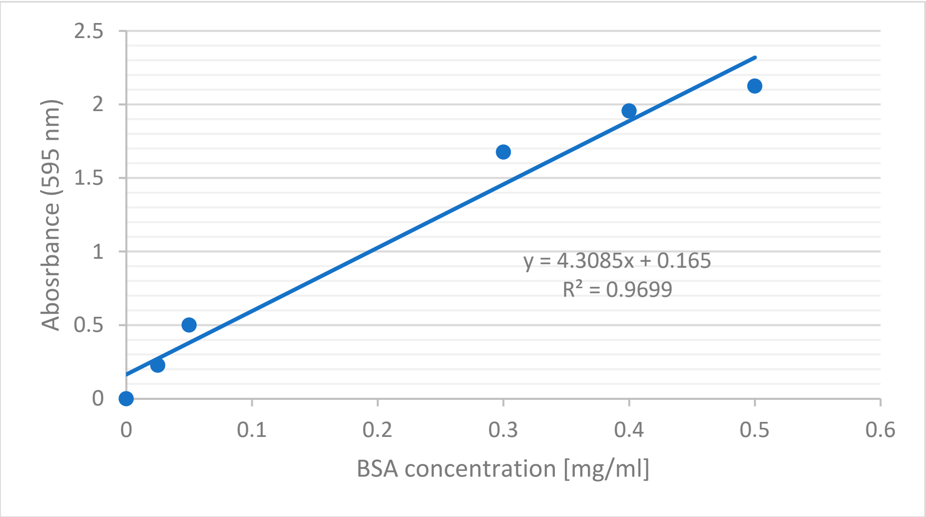

Supplement: Supplementary file 1 [file molecules-28-03085-s001.zip › molecules-2280757-supplementary.pdf]
